# Comparison of the outcome between immunotherapy alone or in combination with chemotherapy in EGFR-mutant non-small cell lung cancer

**DOI:** 10.1038/s41598-021-95628-w

**Published:** 2021-08-09

**Authors:** Chia-I Shen, Heng-Sheng Chao, Tsu-Hui Shiao, Chi-Lu Chiang, Hsu-Ching Huang, Yung-Hung Luo, Chao-Hua Chiu, Yuh-Min Chen

**Affiliations:** 1 Department of Chest Medicine, Taipei Veterans General Hospital, 201, Section 2, Shih-Pai Road, Taipei, 11217 Taiwan; 2 School of Medicine, National Yang Ming Chiao Tung University, 155, Section 2, Linong Street, Taipei, Taiwan; 3 Institute of Clinical Medicine, National Yang Ming Chiao Tung University, 155, Section 2, Linong Street, Taipei, Taiwan

**Keywords:** Cancer therapy, Lung cancer, Oncogenes, Tumour immunology, Oncology

## Abstract

Whether ICIs combined with chemotherapy can improve outcomes in *EGFR*-mutant non-small cell lung cancer (NSCLC) remains uncertain. Patients with EGFR-mutant NSCLC and who progressed on first-line EGFR-TKIs treatment were retrospectively collected. We reviewed the outcome of these patients treated with ICIs or ICIs combined chemotherapy (ICI + C). Total 30 patients were included. The ORR were 9.1% and 25.0% for the ICI and ICI + C groups. The ICI + C group showed the trend of longer progression-free survival and overall survival periods. Patients without the T790M mutation had a significantly longer PFS than did those without this mutation (4.23 [95% CI: 2.75–5.72] vs. 1.70 [95% CI: 0.00–3.51] months, HR:4.45, *p* = 0.019). ICIs combined with chemotherapy tended to be more effective than ICIs alone in pretreated *EGFR*-mutant NSCLC. The T790M mutation may be a potential biomarker.

## Introduction

The treatment of non–small cell lung cancer (NSCLC) has been transformed considerably in recent decades. Epidermal growth factor receptor tyrosine kinase inhibitors (EGFR-TKIs) have been used as an effective treatment for *EGFR*-mutant NSCLC^1^. Compared with traditional chemotherapy, EGFR-TKI treatment is associated with higher response rates and prolonged survival. Nonetheless, most patients typically experience disease progression after 9–14 months of EGFR-TKI treatment^2–6^, primarily due to acquired resistance, which remains the main clinical challenge. Consequently, third-generation EGFR-TKIs have been developed to overcome TKI resistance, particularly in the driver oncogene T790M^5,6^. For patients without the T790M mutation or other resistance mechanisms, chemotherapy remains the standard subsequent treatment^7,8^. Nevertheless, therapeutic options are limited for patients once treatment with TKIs has been exhausted^9^.

Immune checkpoint inhibitors (ICIs) are emerging as novel therapeutic modalities for various malignancies. ICIs such as anti–programmed death 1 (PD-1) and anti–programmed death-ligand1 (PD-L1) have been approved for first- and second-line treatment of NSCLC. ICIs treatment as monotherapy or in combination with chemotherapy has been associated with greater survival compared with chemotherapy alone^10–12^. However, the administration of ICIs in *EGFR*-mutant cohorts has been reported to have suboptimal outcomes^13–16^. Serial clinical trials and meta-analyses have suggested that treatment with chemotherapy alone engendered higher survival in patients with *EGFR* mutations than did treatment with ICIs alone^8,17^. To overcome this obstacle, the administration of ICIs combined with chemotherapy has been associated with favorable outcomes in patients harboring *EGFR* mutations^18–20^. The IMpower150 study and PROLUNG study showed the clinical benefits of combination therapy in the *EGFR*-mutant population^20,21^. However, a direct comparison of late-line immune monotherapy and combination therapy in patients with oncogene variations is lacking. Furthermore, although patients selected in previous trials may have clinical benefits, information is lacking with regard to optimal predictive biomarkers^22–25^.

Accordingly, we conducted a retrospective study to compare the efficacy and clinical outcomes of treatment with ICIs alone and with ICIs in combination with chemotherapy in patients with metastatic *EGFR*-mutant NSCLC. Our objective was to demonstrate real-world applications of immunotherapy combined with chemotherapy in *EGFR*-mutant NSCLC.

## Materials and methods

### Patient cohort

This retrospective observational study was conducted in a tertiary medical center. We enrolled patients with stage IV *EGFR*-mutant NSCLC who received immunotherapy alone or immunotherapy in combination with chemotherapy as their subsequent treatment after disease progression. All patients had been treated with first- or second-generation EGFR-TKIs. We excluded patients with incomplete medical records. In addition, we excluded patients who received a combination of ICIs and anti- vascular endothelial growth factor (VEGF) inhibitors (e.g., bevacizumab) without any chemotoxicity agents. Data were collected between January 2014 and December 2019. We used the staging system described in the 7th edition of the American Joint Committee on Cancer.

### Study design

We collected information on the patients’ clinical characteristics such as sex, smoking history, age, performance status (Eastern Cooperative Oncology Group [ECOG]), *EGFR*-mutation status, cancer staging, and treatment lines through a chart review. We divided the enrolled patients into two treatment groups: the ICI group, comprising those who received ICIs alone as their subsequent treatment, and ICI + C group, comprising those who received ICIs along with chemotherapy. The *EGFR*-mutation profile of each patient was obtained from a chart review. *EGFR* mutations were detected through the cobas EGFR Mutation Test v2. (Roche Molecular Systems Inc., Pleasanton, CA) using tissue samples or liquid biopsy. We analyzed tumor PD-L1 expression by using a PD-L1 IHC 22C3 assay (Dako, Carpinteria,

CA). The ICIs were anti-PD-1/PD-L1 antibodies, including nivolumab, pembrolizumab, atezolizumab, and durvalumab. We calculated progression-free survival (PFS) as the interval from the date of the treatment to the date of disease progression or death. We calculated overall survival (OS) as the interval from the date of the treatment to the date of death or last follow-up. The treatment response was assessed by a clinical physician according to Response Evaluation Criteria in Solid Tumors analysis (RECIST ver 1.1). All experiments were performed in accordance with the relevant guidelines and regulations. This study was approved and the informed consent was waived by the Institutional Review Board of Taipei Veterans General Hospital (2020-07-046CC).

### Statistical analysis

We compared patients’ characteristics by using Pearson’s chi-square test or Fisher’s exact test. We performed a survival analysis using Kaplan–Meier survival curves for PFS and OS. The Cox proportional hazard regression model and logistic regression were applied to analyze clinical features and outcomes. A *p* value of < 0.05 was considered to be statistically significant, and all *p* values were two-sided. We used SPSS software (version 24.0, IBM Corp., Armonk, NY, USA) for all statistical analyses.

### Ethical disclosure

This study was approved by the Institutional Review Board of Taipei Veterans General Hospital (2020-07-046CC).

## Results

### Patient characteristics

This study included a total of 30 patients. The median age was 66.5 years (45–85 years). Approximately 86.7% of the patients were never smokers, and 43.3% of the patients were men. Most of the patients (83.3%) had a favorable performance status (ECOG: 0–1). All patients had stage IV NSCLC with adenocarcinoma confirmed by histology. Moreover, all patients possessed *EGFR* mutations, with 56.7% having exon 19 deletion, 30.0% having an L858R mutation, and 13.3% having another uncommon mutation. The PD-L1 assay had been conducted for only 20% of the patients. The median number of treatment lines before ICIs therapy was 4^3–11^. All patients had been treated with first- or second-generation EGFR-TKIs and subsequent chemotherapy. The objective response rate (ORR) was 76.7%. The median PFS for EGFR-TKIs was 12.20 months (95% confidence interval [CI]: 8.99–15.41). Before subsequent ICIs treatment, 53.3% of the patients had brain metastasis, and 10% of the patients had liver metastasis. Considering immunotherapy, 22 patients received immunotherapy alone (ICI group) and 8 patients received immunotherapy in combination with chemotherapy (ICI + C group). We observed no significant difference between the two treatment groups at baseline. Table 1 presents a summary of the characteristics. Different ICIs were used, including nivolumab (50.0%), pembrolizumab (6.7%), atezolizumab (10.0%), and durvalumab (6.7%). Moreover, 23.3% of the patients received nivolumab combined with chemotherapy, and 3.3% received pembrolizumab with chemotherapy (Supplementary Table 1).

**Table 1:** Patients with EGFR mutations (n = 30).

Patient characteristics (%)	All patients (n = 30)	ICI (n = 22)	ICI + C (n = 8)	*p* value
**Sex**				
Male	13 (43.3)	10 (45.5)	3 (37.5)	1.000
Female	17 (56.7)	12 (54.5)	5 (62.5)	
**Median age**	66.5 (45–85)	65.5 (45–78)	67.5 (55–85)	0.393
**Smoking**				
Never smoker	26 (86.7)	18 (81.8)	8 (100)	0.550
Ever smoker	4 (13.3)	4 (18.2)	0 (0)	
**ECOG**				
0–1	25 (83.3)	18 (81.8)	7 (87.5)	1.000
≥ 2	5 (16.7)	4 (18.2)	1 (12.5)	
**Median of previous treatment lines**	4 (3–11)	5.5 (3–11)	4 (3–6)	0.185
**EGFR mutation**				
Exon 19 deletion	17 (56.7)	13 (59.1)	4 (50.0)	0.848
L858R	9 (30.0)	6 (27.3)	3 (37.5)	
Uncommon mutation*	4 (13.3)	3 (13.6)	1 (12.5)	
**Liver metastasis**				
Liver mets( −)	27 (90.0)	22 (100)	5 (62.5)	0.014
Liver mets( +)	3 (10.0)	0 (0)	3 (37.5)	
**Brain metastasis**				
Brain mets( −)	14 (46.7)	11 (50.0)	3 (37.5)	0.689
Brain mets( +)	16 (53.3)	11 (50.0)	5 (62.5)	

*One exon 20 insertion, two G719X, one L861Q.

### Treatment outcomes of immunotherapy and combination chemotherapy

**Figure 1:**
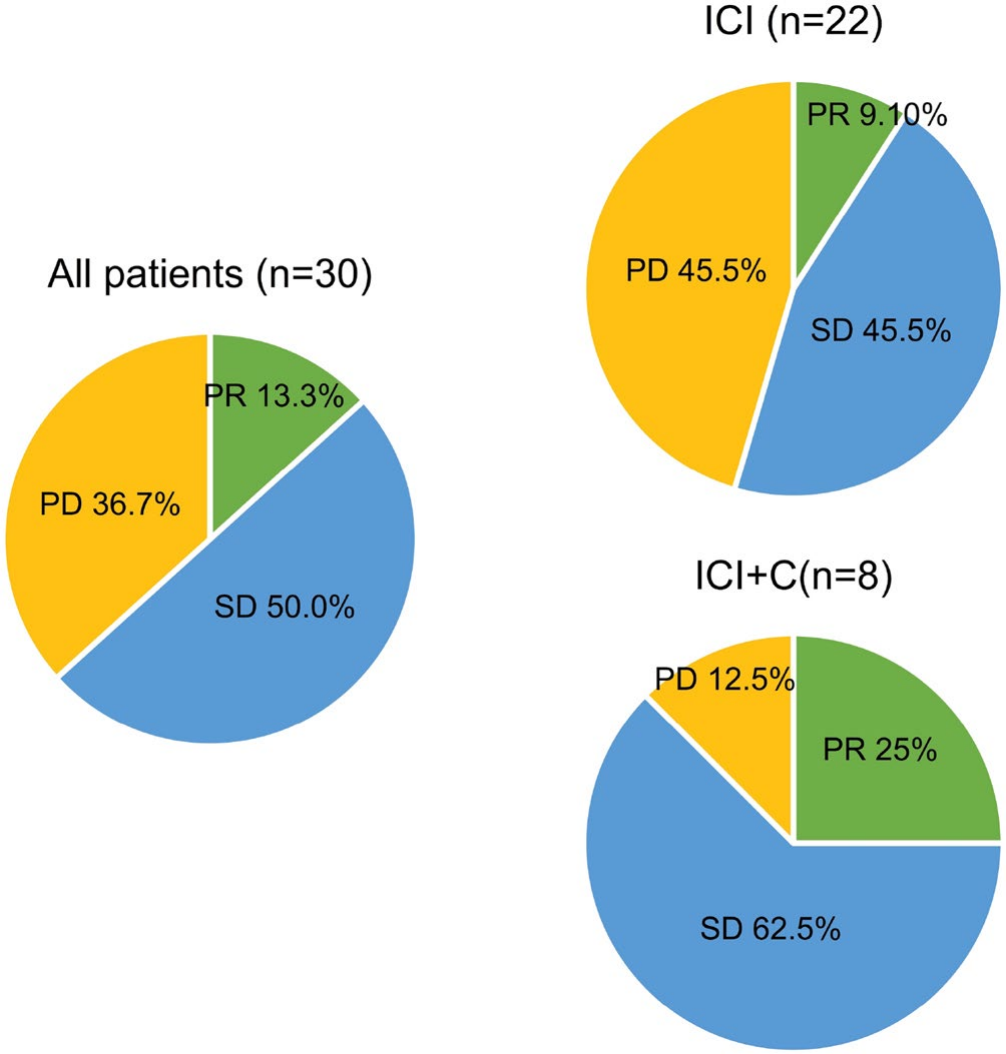
Treatment response to ICI and ICI + C.

**Figure 2:**
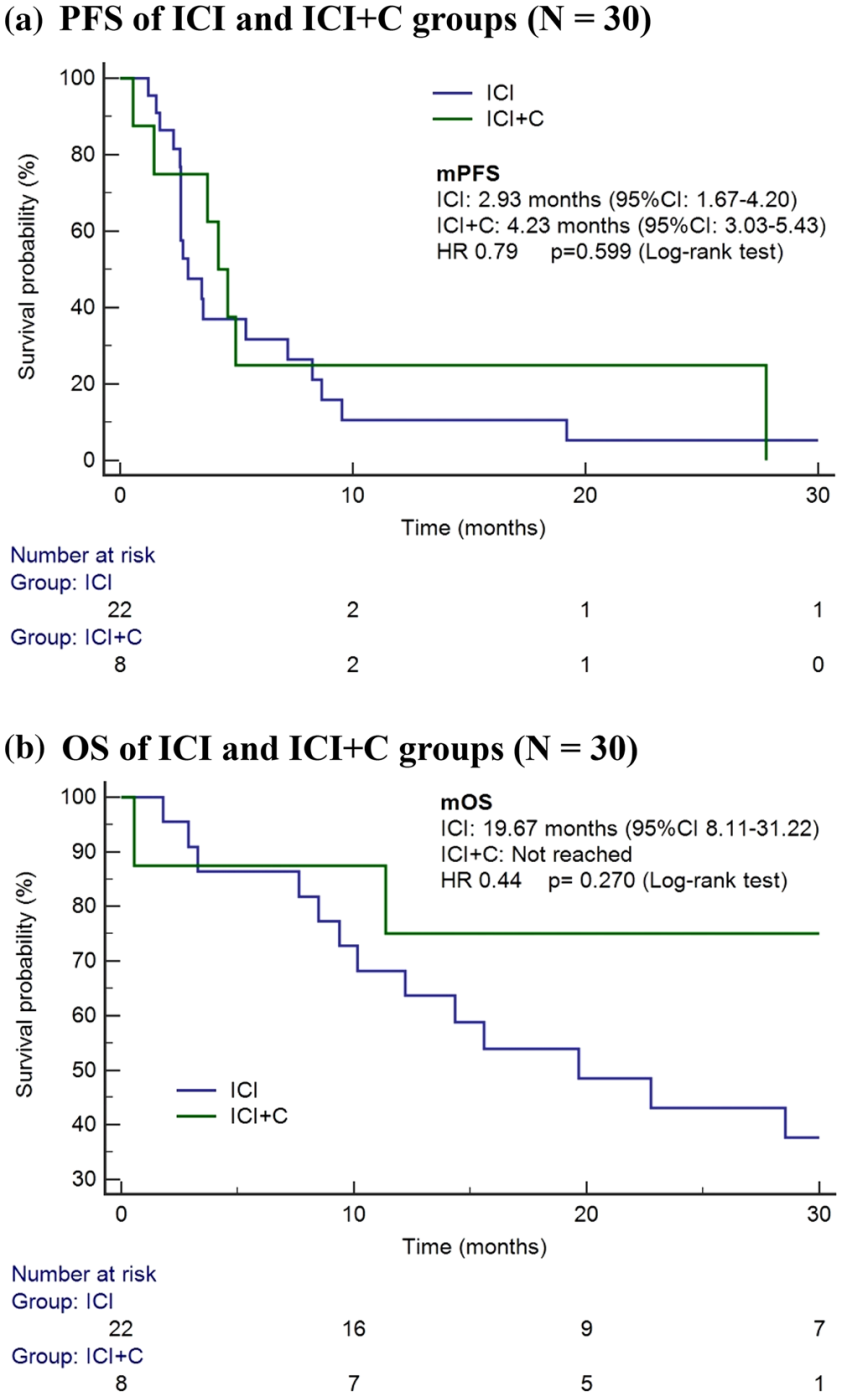
(**a**) PFS of ICI and ICI + C groups (n = 30). (**b**) OS of ICI and ICI + C groups ( n= 30).

The ORRs in the ICI and ICI + C groups were 9.1% and 25.0%, respectively. Furthermore, the disease control rates in the ICI and ICI + C groups were 54.6% and 87.5%, respectively (Fig. 1; Supplementary Table 2). Among the 30 patients, only 8 had documented immunotherapy-related toxicity, which ranged from grade 1 to 2 (skin rash, liver enzyme elevation, fatigue). The median follow-up period of this cohort was 16.76 months (95% CI: 8.49–25.04). The median PFS was 3.57 months (95% CI: 2.18–4.95) and OS was 22.77 months (95% CI: 7.18–38.36) of this study cohort. To divide the patients into two groups by their treatment: the ICI + C group had a slightly longer PFS period than did the ICI group (4.23 [95% CI: 3.03–5.43] vs. 2.93 [95% CI: 1.67–4.20] months; *p* = 0.599; Fig. 2A). In addition, the ICI + C group tended to have a longer OS period than did the ICI group (not reached vs. 19.67 [95% CI: 8.11–31.22] months; *p* = 0.270; Fig. 2B).

### Association between clinical significance and treatment outcomes

Among the 30 patients with *EGFR* mutations who were subsequently treated with immunotherapy or combination chemotherapy, those with liver metastasis had a higher risk of disease progression than did those without liver metastasis (hazard ratio [HR]: 11.07, 95% CI: 1.11–110.48; *p* = 0.041). Other clinical factors, including age, sex, smoking history, performance status, and brain metastasis, were not associated with PFS. A higher number of previous treatment lines was not associated with a higher risk of progression. We used the median PFS for prior EGFR-TKI treatment as the cutoff value and observed that the PFS for prior EGFR-TKI treatment was not associated with the PFS for ICIs treatment. After adjustment for clinical characteristics, the ICI + C group was associated with a longer PFS period compared with the ICI group (HR: 0.24, 95% CI: 0.06–0.97; *p* = 0.045; Table 2). However, factors such as liver metastasis (HR: 0.968, 95% CI: 0.04–32.28; *p* = 0.968) and combination chemotherapy (HR: 0.586, 95% CI: 0.05–5.32; *p* = 0.586) were not statistically associated with OS. Only the performance status of patients was significantly associated with OS; patients with poor status had shorter OS (HR: 24.09, 95% CI: 3.70–157.06; *p* = 0.001; Table 3).

**Table 2:** Cox regression of factors related to PFS (n= 30).

	Univariate analysis			Multivariate analysis		
	Hazard ratio	95% CI	*p* value	Hazard ratio	95% CI	*p* value
Age	1.00	0.96–1.05	0.992	1.03	0.96–1.09	0.431
Female	1.13	0.51–2.50	0.760	2.07	0.54–8.02	0.291
Smoking history	1.42	0.42–4.77	0.573	1.50	0.29–7.72	0.626
ECOG ≥ 2	2.60	0.70–9.75	0.156	3.41	0.77–15.02	0.105
Previous treatment lines > 4	0.89	0.41–1.95	0.773	0.59	0.21–1.64	0.312
Median PFS of prior EGFR-TKI over 12 months	0.84	0.39–1.82	0.660	0.61	0.21–1.77	0.366
Uncommon mutation	0.52	0.15–1.88	0.319	0.42	0.07–2.51	0.345
Brain metastasis	0.94	0.44–2.04	0.877	1.20	0.41–3.55	0.743
Liver metastasis	1.97	0.56–6.91	0.288	11.07	1.11–110.48	0.041
Combination chemotherapy	0.79	0.33–1.91	0.604	0.24	0.06–0.97	0.045

**Table 3:** Cox regression of factors related to OS (n = 30).

	Univariate analysis			Multivariate analysis		
	Hazard ratio	95% CI	*p* value	Hazard ratio	95% CI	*p* value
Age	0.98	0.93–1.03	0.429	0.98	0.92–1.05	0.602
Female	0.98	0.36–2.66	0.966	0.91	0.19–4.38	0.910
Smoking history	1.54	0.44–5.46	0.501	1.77	0.21–14.83	0.598
ECOG ≥ 2	14.61	4.03–52.99	<0.001	24.09	3.70–157.06	0.001
Previous treatment lines > 4	1.95	0.72–5.31	0.190	1.08	0.24–4.79	0.921
Median PFS of prior EGFR-TKI over 12 months	0.93	0.34–2.51	0.881	0.58	0.16–2.11	0.406
Uncommon mutation	0.29	0.04–2.29	0.242	0.15	0.01–1.72	0.128
Brain metastasis	1.54	0.57–4.14	0.395	3.32	0.86–12.85	0.082
Liver metastasis	0.82	0.11–6.35	0.852	1.07	0.04–32.28	0.968
Combination chemotherapy	0.44	0.10–1.97	0.283	0.53	0.05–5.32	0.586

### T790M mutation and treatment response

**Figure 3:**
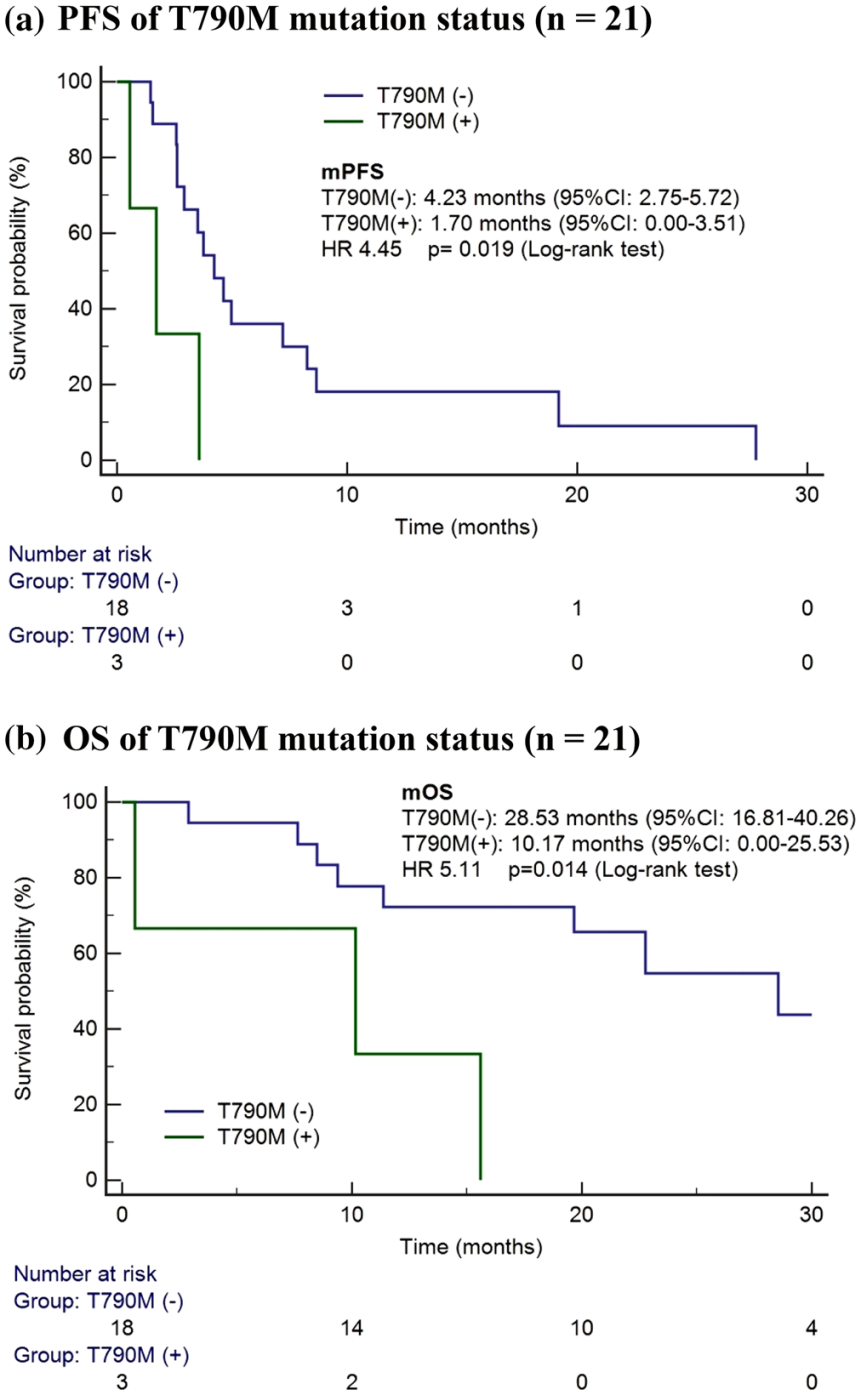
(**a**) PFS of T790M mutation status (n = 21). (**b**) OS of T790M mutation status (n = 21).

We then analyzed the patients for their *EGFR* mutation status and their treatment response to immunotherapy. We re-evaluated a total of 21 patients for their *EGFR* mutation profile (re-biopsy tissue or liquid biopsy) before ICIs treatment. These patients were divided into a T790M-positive group, namely T790M(+), and a T790M-negative group, namely T790M(−). Eighteen patients had no T790M mutation, and only 3 patients were found to be T790M positive. The baseline characteristics are presented in Supplementary Table 3, revealing no obvious difference between the two mutation groups. The PFS period was significantly longer in the T790M(−) group than in the T790M(+) group (4.23 [95% CI: 2.75–5.72] vs. 1.70 [95% CI: 0.00–3.51] months; *p* = 0.019; Fig. 3A). Furthermore, the T790M( −) group had a longer OS period than did the T790M( +) group (28.53 [95% CI: 16.81–40.26] vs. 10.17 [95% CI: 0.00–25.53] months; *p* = 0.014; Fig. 3B). Multivariate analysis also revealed that patients with T790M had greater risk of disease progression (HR: 35.46, 95% CI: 3.18–395.41; *p* = 0.004). The superior treatment response observed for front-line EGFR-TKI treatment (PFS longer than 12 months) was associated with the longer PFS for ICI treatment, regardless of whether combination chemotherapy was administered (HR: 0.16, 95% CI: 0.03–0.80; *p* = 0.025; Supplementary Table 4). These associations among clinical factors and prognosis were not noted in the OS analysis (Supplementary Table 5).

## Discussion

We present the results of patients with *EGFR*-mutant NSCLC who were treated with ICIs or ICIs combined with chemotherapy in a real-world setting. We found that ICIs in combination with chemotherapy showed a trend of superior efficacy along with acceptable treatment response compared with monotherapy involving ICIs. These results are consistent with those reported by previous studies^18–20^. Several possible mechanisms underlie the unsatisfactory efficacy of ICI therapy in patients with *EGFR* mutations. Patients with *EGFR* mutations have lower tumor mutation burden (TMB) levels and reduced tumor-infiltrating lymphocytes compared with other patients; they thus have lower immunogenicity and antitumor immunity^22,23^. The application of TKIs may affect PD-L1 expression and the tumor microenvironment^25–29^, both of which may have an adverse effect on the efficacy of immunotherapy. For example, a study proposed that chemotherapy may contribute to an increase in neoantigens and boost the reaction to immunotherapy^30^. Kuo et al. demonstrated that compared with ICI therapy alone, combining ICI therapy with chemotherapy improved survival in patients with NSCLC^18^. We confirmed this beneficial effect of combination chemotherapy with ICIs in the *EGFR*-mutant population. Cox regression analysis revealed improved PFS for the ICI + C group compared with the ICI group (HR: 0.24, 95% CI: 0.06–0.97; *p* = 0.045). This finding demonstrates a synergistic interaction between chemotherapy and ICIs. For clinicians, this study demonstrated that ICI alone in *EGFR*-mutant NSCLC has poor outcome. Concomitant chemotherapy may improve the benefits of immunotherapy, however, the optimal strategy for such combinations requires further investigation.

We evaluated the possible clinical characteristics affecting outcomes. After adjusting for age, sex, smoking history, performance status, and uncommon mutations, we observed that patients with liver metastasis may have poorer PFS than did those without such metastasis. A previous study also indicated that liver metastasis is an issue affecting immunotherapy because ICIs alone provide minimal therapeutic benefits^31^. This might be associated with the specific microenvironments and immunoregulation of the liver^20,32^. We observed that patients with *EGFR*-mutant NSCLC and liver metastasis showed inferior PFS. By analyzing our data, we identified that in late-line settings, liver metastasis was predictive of poor response. However, not all clinical factors were associated with OS; only poor ECOG showed any predictive value for patient survival. This might be because the patients were all heavily pretreated and the small number of study groups caused no significant OS results. Nevertheless, one can reasonably assume that the performance status may be useful for predicting OS in these patients, given the complicated condition of patients in this late-line setting.

To identify patients that may have benefited from therapy, several biomarkers have been studied for ICIs in *EGFR*-positive cohorts. Smoking status have been reported as a clinical predictor of response to ICIs in *EGFR*-mutant NSCLC^33^. A previous cohort study reported that T790M-negative patients may benefit from ICI treatment after TKI failure^25^. Yamada et al. also demonstrated that uncommon mutations and the absence of T790M mutations are predictive of positive ICIs outcomes^24^. A study on the IMMUNOTARGET cohort also supported the finding that different subtypes of *EGFR* mutations have different PFS periods after ICI treatment^34^. Lau et al. also reported the significant difference of treatment response to ICIs between common and uncommon mutations in retrospective study^35^. Patients with uncommon mutations may have a higher TMB^16^. In addition, patients showing acquired resistance not engendered by T790M mutations are likely to exhibit high PD-L1 levels^24,25^. High PD-L1 expression may be result from the activation of other alternative oncogenic pathway and these pateints may benefit from ICIs administration^36^. Our study confirmed that T790M remains a poor prognostic marker not only for ICIs alone but also for ICIs combined with chemotherapy. In addition, a longer duration of treatment with first-line EGFR-TKIs was associated with a longer PFS, but this finding is not consistent with previous reports. Ichihara et al. reported that the PFS for prior treatment with EGFR-TKIs was negatively associated with that for prior treatment with ICIs^37^. Liu et al. also reported a better response to subsequent immunotherapy in *EGFR*-mutant NSCLC with shorter PFS during EGFR-TKIs treatment^38^. They also conducted single cell RNA-sequencing to prove the different tumor microenvironment between longer and shorter first-line TKI treatment groups. Comparing to their study, our cohort focusing on those with late-line treatment group. Our patients may receive more lines of treatment after EGFR-TKIs failure. In this cohort, tumor mutation loads were more strongly affected by T790M mutation than by previous EGFR-TKI treatment response. Therefore, for PFS, prior treatment with EGFR-TKIs was less informative than T790M as a biomarker under clinical circumstances.

Our study has several limitations. First, the retrospective observational cohort study design has inherent restrictions on the data available for analysis. For example, some molecular profiles, such as PD-L1 expression, was evaluated only in a subset of patients. This was probably due to PD-L1 expression was not extensively applied in the late-line settings. However, PD-L1 expression has been reported to be a significant predictor of ICIs efficacy for *EGFR*-mutated NSCLC patients in the subgroup analysis of the ATLANTIC study^39^. Further investigation focusing on the role of PD-L1 expression in ICIs combined chemotherapy may offer more information. Similarly, there was no data of TMB reported. Second, the total sample size was small. Thirdly, the chemotherapy regimens in our study were heterogeneous, including single agent navelbine, docetaxel, and doublet combining navelbine plus gemcitabine (Supplement Table 1). One patient received paclitaxel, carboplatin and bevacizumab. The heterogenicity was due to the lack of standard-of-care in late-line treatment. Meanwhile, it also reflected the unmet need in clinical practice. Finally, the previous treatment pathways and chemotherapy combinations may have differed among patients. Nevertheless, this is the first study examining the efficacy and outcomes of ICIs administered alone and in combination with chemotherapy in patients with *EGFR*-mutant NSCLC in an area with a high prevalence of *EGFR* mutations.

## Conclusion

ICIs combined with chemotherapy may be more effective and beneficial than ICIs alone in pretreated patients with *EGFR*-mutant NSCLC in the real world. Furthermore, the T790M mutation can be used as a predictive biomarker for poor response to treatments comprising both ICIs alone and ICIs combined chemotherapy.

## Supplementary Information

Supplementary Information.

## Data Availability

The datasets generated during and/or analysed during the current study are not publicly available due to patients’ privacy but are available from the corresponding author on reasonable request.
