# Supplementary material for: Comparison of the outcome between immunotherapy alone or in combination with chemotherapy in EGFR-mutant non-small cell lung cancer
Source: Sci Rep. 2021 Aug 9;11:16122. doi: 10.1038/s41598-021-95628-w (PMC8352947; doi:10.1038/s41598-021-95628-w)
Supplement: Supplementary file 1 — Supplementary Information. [file 41598_2021_95628_MOESM1_ESM.docx]

**Supplement Table 1 PD1 and antiPDL1 used in this cohort (n=30)**

| Drug | Patients (%) |
| --- | --- |
| Atezolizumab | 3 (10.0) |
| Durvalumab | 2 (6.7) |
| Nivolumab | 15 (50.0) |
| Pembrolizumab | 2 (6.7) |
| Nivolumab+chemotherapy* | 7 (23.3) |
| Pembrolizumab+chemotherapy** | 1 (3.3) |

*chemotherapy regimen including navelbine, navelbine+gemcitabine, docetaxel, paclitaxel, paclitaxel+carboplatin+bevacizumab

**chemotherapy regimen: navelbine

**Supplementary Table 2 Response rate of ICI alone and ICI+C (n=30)**

|  | Objective response rate (%) | Disease control rate (%) | P value |
| --- | --- | --- | --- |
| ICI group | 9.1 | 54.6 | 0.159 |
| ICI+C group | 25.0 | 87.5 |  |
| All | 13.3 | 63.3 |  |

**Supplement Table 3 Patients with T790M (n=21)**

| Patient characteristics(%) | All patients (n=21) | T790M(-) (n=18) | T790M (+) (n=3) | P value |
| --- | --- | --- | --- | --- |
| Sex |  |  |  |  |
| Male | 8 (38.1) | 7 (38.9) | 1 (33.3) | 1.000 |
| Female | 13 (61.9) | 11 (61.1) | 2 (66.7) |  |
| Median age | 64 (45-85) | 64 (45-85) | 71 (59-82) | 0.412 |
| Smoking |  |  |  |  |
| Never smoker | 18 (85.7) | 15 (83.3) | 3 (100) | 1.000 |
| Ever smoker | 3 (14.3) | 3 (16.7) | 0 (0) |  |
| ECOG |  |  |  |  |
| 0-1 | 19 (90.5) | 17 (94.4) | 2 (66.7) | 0.271 |
| >=2 | 2 (9.5) | 1 (5.6) | 1 (33.3) |  |
| Median of previous treatment lines | 4 (3-11) | 4 (3-10) | 5 (4-11) | 0.262 |
| EGFR mutation |  |  |  |  |
| Exon 19 deletion | 12 (57.1) | 11 (61.1) | 1 (33.3) | 0.298 |
| L858R | 7 (33.3) | 6 (33.3) | 1 (33.3) |  |
| Uncommon mutation* | 2 (9.5) | 1 (5.6) | 1 (33.3) |  |
| Liver metastasis |  |  |  |  |
| Liver mets(-) | 18 (85.7) | 16 (88.9) | 2 (66.7) | 0.386 |
| Liver mets(+) | 3 (14.3) | 2 (11.1) | 1 (33.3) |  |
| Brain metastasis |  |  |  |  |
| Brain mets(-) | 7 (33.3) | 5 (27.8) | 2 (66.7) | 0.689 |
| Brain mets(+) | 14 (67.7) | 13 (72.2) | 1 (33.3) |  |

*one G719X, one L861Q

**Supplement Table 4 Cox regression of factors related to PFS (n=21)**

|  | Univariate analysis | | | Multivariate analysis | | |
| --- | --- | --- | --- | --- | --- | --- |
|  | Hazard ratio | 95% CI | P value | Hazard ratio | 95% CI | P value |
| Age | 0.99 | 0.94-1.04 | 0.670 | 1.04 | 0.95-1.13 | 0.419 |
| Female | 0.48 | 0.17-1.37 | 0.169 | 0.36 | 0.05-2.58 | 0.308 |
| Smoking history | 1.04 | 0.23-4.64 | 0.961 | 0.76 | 0.05-11.34 | 0.840 |
| ECOG>=2 | 2.51 | 0.30-21.02 | 0.395 | 0.21 | 0.00-19.24 | 0.499 |
| Tx lines>4 | 1.07 | 0.42-2.72 | 0.891 | 0.37 | 0.08-1.79 | 0.216 |
| TKI>=12months | 0.45 | 0.17-1.24 | 0.122 | 0.16 | 0.03-0.80 | 0.025 |
| T790M | 4.45 | 1.14-17.44 | 0.032 | 35.46 | 3.18-395.41 | 0.004 |
| BM | 1.91 | 0.67-5.40 | 0.226 | 2.04 | 0.44-9.54 | 0.365 |
| Liver mets | 2.12 | 0.57-7.90 | 0.262 | 4.27 | 0.18-100.88 | 0.368 |
| Combined chemo | 0.65 | 0.24-1.74 | 0.386 | 0.28 | 0.03-2.52 | 0.255 |

**Supplement Table 5 Cox regression of factors related to OS (n=21)**

|  | Univariate analysis | | | Multivariate analysis | | |
| --- | --- | --- | --- | --- | --- | --- |
|  | Hazard ratio | 95% CI | P value | Hazard ratio | 95% CI | P value |
| Age | 0.97 | 0.92-1.02 | 0.259 | 1.00 | 0.87-1.14 | 0.964 |
| Female | 0.33 | 0.10-1.11 | 0.073 | 0.001 | 0.00-7.90 | 0.134 |
| Smoking history | 0.35 | 0.09-1.34 | 0.125 | 5.48 | 0.03-1182.20 | 0.535 |
| ECOG>=2 | 1062257.084 | 0.00- 2.300E+170 | 0.943 | 785447.85 | 0.00- 6.066E+196 | 0.952 |
| Tx lines>4 | 1.57 | 0.50-4.94 | 0.440 | 0.35 | 0.01-13.18 | 0.571 |
| TKI>=12months | 0.19 | 0.05-0.71 | 0.013 | 0.002 | 0.00-6.49 | 0.130 |
| T790M | 5.11 | 1.21-21.61 | 0.026 | 1494.24 | 0.32- 6946428.466 | 0.090 |
| BM | 1.38 | 0.41-4.60 | 0.605 | 0.82 | 0.05-12.52 | 0.889 |
| Liver mets | 0.83 | 0.10-6.63 | 0.856 | 0.01 | 0.00-2339.62 | 0.454 |
| Combined chemo | 2.69 | 0.57-12.64 | 0.210 | 0.60 | 0.01-52.02 | 0.823 |
